# Lipase Assisted (*S*)-Ketoprofen Resolution from Commercially Available Racemic Mixture

**DOI:** 10.3390/ph14100996

**Published:** 2021-09-29

**Authors:** Daniela Estrada-Valenzuela, Víctor H. Ramos-Sánchez, Gerardo Zaragoza-Galán, Jose C. Espinoza-Hicks, Alejandro Bugarin, David Chávez-Flores

**Affiliations:** 1 Facultad de Ciencias Químicas, Universidad Autónoma de Chihuahua, Circuito Universitario, Chihuahua 31125, Mexico; p291826@uach.mx (D.E.-V.); vramos@uach.mx (V.H.R.-S.); gzaragoza@uach.mx (G.Z.-G.); jhicks@uach.mx (J.C.E.-H.); 2 Department of Chemistry & Physics, Florida Gulf Coast University, Fort Myers, FL 33965, USA

**Keywords:** enantiomer, resolution, biocatalyst, enzymatic, ketoprofen, (*S*)-ketoprofen, chiral

## Abstract

Ketoprofen is a commercially available drug sold as a racemic mixture that belongs to the family of non-steroidal anti-inflammatory drugs known as profens. It has been demonstrated (in vitro) that (*S*)-ketoprofen is around 160 times more potent than its enantiomer (*R*)-ketoprofen, while accumulation of (*R*)-ketoprofen can cause serious side effects, such as dyspepsia, gastrointestinal ulceration/bleeding, pain, salt and fluid retention, and hypertension. In this work, four commercially available lipases were systematically assessed. Parameters such as conversion, enantiomeric excess, and enantioselectivity were considered. Among them, and by evaluating lipase load, temperature, solvent, and alcohol, *Candida rugosa* lipase exhibited the best results in terms of enantioselectivity ***E*** = 185 ((*S*)-enantiopreference) with esterification conversions of ***c*** = 47% (out of 50%) and enantiomeric excess of 99%. The unreacted (*R*)-enantiomer was recovered by liquid-liquid extraction and racemized under basic media, which was recycled as starting material. Finally, the (*S*)-alkyl ketoprofen ester was successfully enzymatically hydrolyzed to the desired (*S*)-ketoprofen with ***c*** = 98.5% and 99% ee. This work demonstrated the benefit and efficiency of using *Candida rugosa* lipase to kinetically resolve racemic ketoprofen by an environmentally friendly protocol and with the recycling of the undesired (*R*)-ketoprofen.

## 1. Introduction

Non-steroidal anti-inflammatory drugs (NSAIDs) are a group of important drugs used for their broad anti-inflammatory, antipyretic, and analgesic potential, as well as being popular for not causing sedative addictive or depressive effects [1]. These therapeutic effects are attributed to their inhibition of the enzyme cyclooxygenase (COX), which is involved in the production of leukotrienes, thromboxanes, and prostaglandins: all mediators of inflammation, fever, and pain [2]. There are two isoforms of COX: COX-1 is a constitutively expressed isoform that intervenes in the processes of hemostasis, regulation of the intestinal mucosa, and other basic functions, while COX-2 intervenes in the regulation of the inflammatory process and is an inducible enzyme [3]. Therefore, it is possible to regulate inflammation, pain, and fever through the specific inhibition of COX-2 enzyme, which in turn inhibits the formation of eicosanoids giving the desired therapeutic effects [4,5,6]. Some of the most common drugs within this category are ibuprofen, ketoprofen, acetaminophen, naproxen, acetylsalicylic acid, and diclofenac. Several studies have shown that 68% of self-medication drugs in the United States are NSAIDs [7,8]. Just in United States of America, about 30 billion doses of NSAIDs are ingested per year [9].

Currently, there are many commercial drugs that are racemic mixtures despite the fact that several studies have demonstrated that only one of the two isomers exhibit therapeutic effects [10,11]. The chiral enantiomer that has the desired biological activity is called eutomer, while the dystomer is defined as the enantiomer and may be bio-inert, show lower activity, or present an unwanted bioactivity [12,13]. In 2020, Mwamwitwa and co-workers reported that 60% of total human medicines are chiral. Out of the chiral commercial drugs, 74% are racemic mixtures and only 26% are single enantiomers [14]. Nonetheless, the administration of enantiomeric pure drugs has advantages over the use of racemic ones with respect to efficiency, metabolic effectiveness, and fewer toxicological effects [15]. Ketoprofen 2-(3-benzoylphenyl)-propionic acid is an NSAID that achieves its effect by inhibiting the synthesis of prostaglandins through the enantioselective inhibition of COX. Specifically, ketoprofen has shown high efficiency in the treatment of rheumatoid arthritis and the treatment of osteoarthritis [16]. Ketoprofen is sold as a racemic mixture; several studies, however, have reported that the desired therapeutic effect is attributed to the (*S*)-enantiomer, while the (*R*)-enantiomer is the one that can present unwanted effects in the central nervous, gastrointestinal, and renal systems [14,16,17]. Although some profens have displayed metabolic chiral inversion (e.g., ibuprofen), ketoprofen has shown negligible chiral inversions in humans [18,19]. Studies on the metabolism of ketoprofen have shown that ketoprofen is rapidly absorbed, metabolized, and excreted as glucuronic ester, also known as ketoprofen glucuronide, through urine [20,21]. The most common methods to produce pure or enantiomeric-enriched drugs are asymmetric synthesis, kinetic resolution, crystallization, and chiral chromatography [8]. Nonetheless, enzymatic catalysis, or bio-catalysis, is catching up due to lower production cost, high yields, and a sustainable and environmentally friendly process [22]. Among biocatalysts, lipases play an important role on enantioselective reactions such as hydrolysis, esterification, amidation, thioesterification, etc., as these reactions can be carried out in aqueous media, non-polar solvents, and solvent-free conditions [23,24,25,26,27]. In addition, it is the concentration of water that determines whether lipases can either perform hydrolysis or an esterification, to name an example (a promiscuous biocatalyst of great benefits in the pharmaceutical area) [28]. In the past, some lipases have been successfully applied for the resolution of NSAIDs by enantioselective esterification and hydrolysis [1,2,29]. *Candida rugosa, Mucor javanicus, Candida antarctica*, and porcine pancreatic lipases are intracellular lipolytic enzymes that have shown (*S*)-preference in the resolution of ketoprofen, ibuprofen, suprofen, and naproxen [1,30,31,32]. This work reports an environmentally friendly methodology for the resolution of (*S*)-ketoprofen using lipase (*Candida rugosa*) as the enantioselective biocatalysts. The racemization and recycling of the undesired (*R*)-ketoprofen is also reported.

## 2. Results

### 2.1. Ketoprofen Extraction and Characterization 

**Figure 1:**
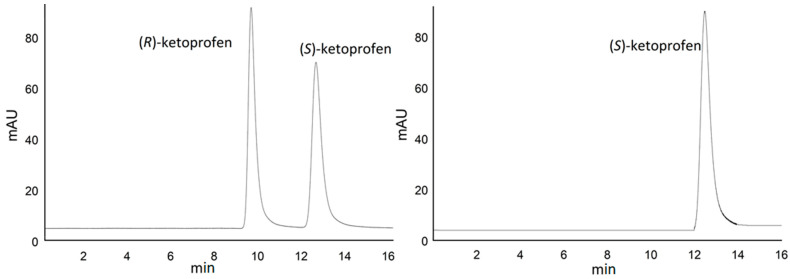
HPLC chromatograms; identification of (*S*)-ketoprofen standard (right) *tr* = 12.67 min and racemic ketoprofen (left) with (*R*)-ketoprofen *tr* = 9.55 min and (*S*)-ketoprofen *tr* = 12.63 min. (*tr* = time retention).

FTIR and ^1^H NMR spectra (Figures S1–S3 of the Supplementary Material) as well as chiral high-performance liquid chromatography (HPLC) chromatograms confirmed that high pure racemic ketoprofen was successfully isolated from over-the-counter and inexpensive pills by solid-liquid extraction with a purity of 99.5%, (m.p. 93–94 °C), and a 98% efficiency based on the expected amount according to the label located on the commercial racemic drug. In order to determine the enantiomers retention time (*rt*) on the HPLC chromatogram, an (*S*)-ketoprofen certified standard was acquired and analyzed. The (*S*)-ketoprofen appeared at 9.55 min *rt*, whereas (*R*)-ketoprofen showed a 12.63 min *rt* (from the racemic mixture, as shown in Figure 1).

### 2.2. Biocatalyst Selection

**Scheme 1:**
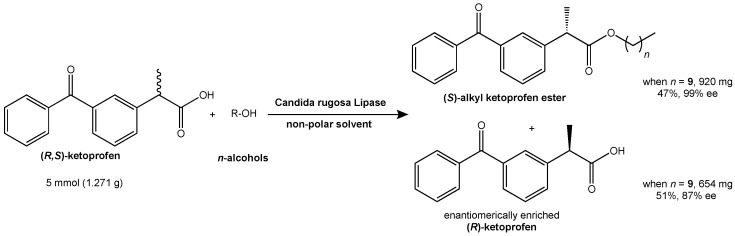
Enantioselective enzymatic ketoprofen esterification reaction. (Lipases from: *Candida rugosa*, *Candida antarctica*, *Mucor javanicus* and porcine pancreas, temperatures of 30, 40, and 50 °C, solvents: hexane, cyclohexane, and isooctane; alcohols: methanol, *n*-butanol, *n*-octanol, and *n*-decanol).

To optimize the reaction conditions for the enantioselective esterification reaction, different biocatalysts were screened as shown on Scheme 1. Likewise, the best temperature and biocatalyst load were studied. For instance, racemic ketoprofen was converted into (*S*)-decyl ketoprofen ester using *Candida rugosa* lipase, *Candida antarctica* lipase type B (Novozym 435), *Mucor javanicus* lipase, and porcine pancreatic lipase as biocatalysts.

*Candida antarctica* lipase (CAL) was quicky discarded due to its very low activity in both conversion (***c***) and enantioselective (ee) during the esterification attempts (data not shown). However, using *Candida rugosa* lipase (CRL) and adopting our previously reported conditions for similar substrates [2]: 250 rpm, 1:1 w:w ratio of substrate:alcohol and cyclohexane as solvent, the highest conversion yield (*c* = 44%) and enantiopreference to the (*S*)-ketoprofen (87% ee) were found. On the other hand, both *Mucor javanicus* lipase (MJL) and porcine pancreatic lipase (PPL) produced the expected adduct, albeit in low yields and modest enantioselectivity (Table 1). It is important to note that reactions stirred for longer than 60 h start showing degradation by-products, which were observed by HPLC (Figure S4, see Supplementary Material), which corroborates D’Antona’s observations [33].

### 2.3. Solvent and Alcohol Selection

Having *Candida rugosa* lipase as the best biocatalyst (Table 1), we then screened several alcohols and three organic solvents for the esterification reaction (Table 2). It was found that *n*-decanol and isooctane are the best combination, as shown in Table 2. From our findings, it was determined that the longer the alkyl chain of the alcohol, the greater the reaction rate and enantioselectivity of the desired product. Longer chain alcohols (>10 carbons) were not tested due to their high cost, making the protocol expensive.

### 2.4. (S)-Decyl Ketoprofen Ester Isolation

**Figure 2:**
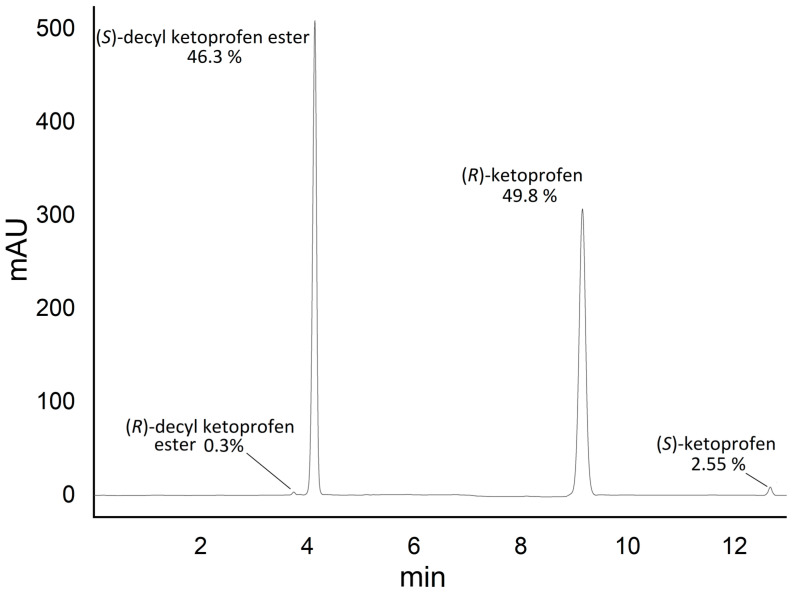
HPLC chromatogram of an esterification reaction at 48 h.

**Figure 3:**
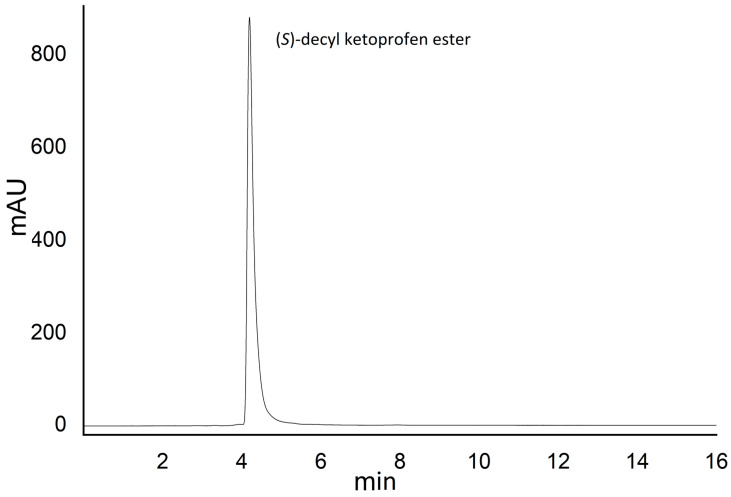
(*S*)-decyl ketoprofen ester isolated chromatogram.

As shown in Figure 2, after 48 h of reaction the esterification reaction reaches the maximum observed conversion (*c* = 46.6% out of 50%). The reaction was stopped, and the enzyme was removed by vacuum filtration. The filtrated was concentrated under reduce pressure (using a standard roto-evaporator) to get crude (*S*)-alkyl ketoprofen ester and the unreacted enriched (*R*)-ketoprofen mixture. The oily solution was then poured into a 125 mL separation funnel. Then, 50 mL of aqueous 10% NaHCO_3_ solution were added, the mixture was vigorously stirred, followed by three extractions using 90 mL of hexanes in total. The hexanes solution was then concentrated under reduced pressure to afford crude (*S*)-decyl ketoprofen ester. Finally, silica-gel flash chromatography (80:20 Hexanes-EtOAc) was used to purify the adduct. The pure adduct was characterized by chiral HPLC (Figure 3), ^1^H NMR, and FTIR (Figures S5 and S6 in Supplementary Material). It is important to note that the aqueous solution contains the enantiomerically enriched (*R*)-ketoprofen that was saved for further racemization.

### 2.5. (R)-Ketoprofen Racemization

After several attempts to induce (*R*)-ketoprofen racemization from the isolated 87% ee, under reflux in acidic conditions (0.1 M sulfuric acid solution) only low racemization was observed (73% ee) even after 24 h. This may be due to the low or null formation of ketoprofen enol. Basic conditions were attempted. For instance, using sodium hydroxide (0.1 M) and reflux for 12 h resulted in a modest racemization of (39% ee). Fortunately, after using a 0.1 M potassium hydroxide solution on a reflux system for 4 h, quantitative conversion was almost achieved, obtaining ketoprofen with (1.6% ee). This is a mixture of 50.8 (*R*)-ketoprofen and 49.2 (*S*)-ketoprofen, practically a racemic mixture. On the other hand, using alternate energy sources such as a microwave (power of 250 W at a temperature of 105 °C) and under acid conditions (0.1 M sulfuric acid solution), racemization was modest (27% ee), and under basic medium (0.1 M KOH or NaOH) racemization (12% ee) was determined. Although this method seemed to be efficient for the racemization reaction, degradation was observed at longer times. The HLPC chromatograms started showing new signals at different retention times, which indicates the generation of new and undesirable chemical compounds (Figure S4). For this reason, the use of microwaves was ruled out for this reaction.

### 2.6. (S)-Decyl Ketoprofen Hydrolysis

**Scheme 2:**
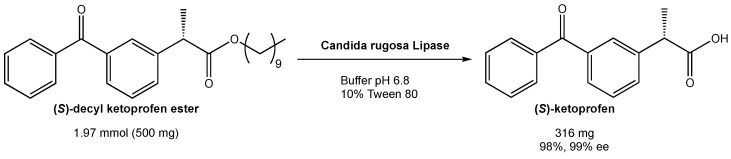
Hydrolysis of (*S*)-decyl ketoprofen to (*S*)-ketoprofen.

Due to the low solubility of (*S*)-decyl ketoprofen ester in aqueous solution, different concentrations of *Candida rugosa* lipase and surfactant (Tween 80) were used for the hydrolysis of the ester in order to increase lipase activity. Using *Candida rugosa* lipase (20%) as a biocatalyst and 10% Tween 80, an almost quantitative conversion (98.59% (±0.175)) of (*S*)-decyl ketoprofen ester to the desired (*S*)-ketoprofen with 99% ee was achieved. Other attempts without surfactant and other variables that were less efficient. These results led us to conclude that the null solubility of the (*S*)-decyl ketoprofen ester in water prevented collisions between the enzyme and the substrate. However, adding Tween 80 as surfactant increased its solubility, as previously observed by Liu and co-workers [5]. This optimal reaction condition allowed high conversion and zero erosion of its initial enantioselectivity (99% ee) for the hydrolysis of (*S*)-decyl ketoprofen ester to pure (*S*)-carboxylic acid (Scheme 2).

## 3. Discussion

Racemic ketoprofen is a widely used over-the-counter drug. (*S*)-ketoprofen shows anti-inflammatory activity, while (*R*)-ketoprofen inhibits the action of (*S*)-ketoprofen. Therefore, a reliable and environmentally friendly method for the resolution of racemic ketoprofen to the active (*S*)-ketoprofen is desired. Thus far, lipases have been used to resolve many racemic mixtures, including ketoprofen. However, some of these attempts employ immobilized enzymes (expensive), halogenated solvents, and enantiomeric enrichments (additional steps), and still those methods have room for improvement (in both yields and ee’s) [33]. To the best of our knowledge, this is the first publication that resolves the (*S*)-ketoprofen and reports a method to recycle (*R*)-ketoprofen. Only a few reports of asymmetric synthesis of (*S*)-ketoprofen were found [34,35,36,37]; these techniques employ more complex separation methods and more toxic chemicals. In addition to the simplicity and environmentally friendliness of the protocol, to our knowledge, our work represents the highest overall conversion. We hope that pharmaceutical companies adopt our method to resolve and perhaps commercialize enantiomeric-enriched (*S*)-ketoprofen and stop selling a racemic mixture.

## 4. Materials and Methods

### 4.1. Materials

Racemic ketoprofen was isolated from inexpensive, over-the-counter tablets (100 mg PiSA Farmaceutica). (*S*)-(+)-ketoprofen analytical standard, *Cándida rugosa* lipase, *Cándida antarctica* lipase type B, porcine pancreatic Lipase, and *Mucor javanicus* lipase were obtained from Sigma Aldrich Company (St. Louis, MO, USA). Decan-1-ol, octan-1-ol, butan-1-ol, and methan-1-ol were purchased from Alfa Aesar. Cyclohexane, hexane, hexanes, isopropanol, and isooctane were purchased from Tedia (Ohio, OH, USA). Monobasic potassium phosphate and dibasic potassium phosphate were purchased from Spectrum Chemical Company. All other chemicals and analytical-grade reagents were from commercial sources and used as received.

### 4.2. Ketoprofen Extraction

Racemic ketoprofen was extracted from commercial tablets in a 125 mL Erlenmeyer flask by dissolving 20 tablets in 80 mL of acetone, followed by vigorously stirring for 30 min using a magnetic stir bar. Then, the solution was filtered using paper filters to remove the insoluble coating and fillers. The acetone filtrate mixture was concentrated under reduced pressure, in a rotavapor, to recover racemic ketoprofen in an almost quantitative yield. A rotary evaporator (BÜCHI model R-215, Flawil, Switzerland) was used to remove volatile solvents. The crystals obtained were characterized by melting point (m.p.), proton nuclear magnetic resonance, (^1^H NMR) Fourier transform infrared spectroscopy (FTIR), and chiral high-performance liquid chromatography (HPLC).

### 4.3. High-Performance Liquid Chromatography

High-performance liquid chromatography (HPLC) was performed with a Chiracel OJ chiral column (Diacel Chemical Industries). The HPLC instrument (Dionex, Sunnyvale, CA, USA) was equipped with a Dionex LPG-3400-D quaternary analytical pump, Dionex UltiMate 3000 diode array detector, Dionex solvent degaser, and Chromeleon CM-PCS-1 Software. The mobile phase normally used was hexanes/isopropanol/trifluoroacetic acid (90:9.9/0.1% v/v/v). The UV detector wavelength was set to 254 nm, the flow rate was 1.0 mL/min, and the temperatures of the column and injection compartment were 35 and 20 °C, respectively.

### 4.4. Enzymatic Esterification of Ketoprofen

In a typical reaction of 1.271 g (5 mmol) of racemic ketoprofen, 5 mmol of an alcohol (0.160 g of methanol, 0.371 g of *n*-butanol, 0.651 g of *n*-octanol, or 0.791 g of *n*-decanol), the equivalent amount of lipase corresponding to 20%, 40%, and 60% weight with respect to the total mass of reagents (0.286, 0.573, or 0.859 g when methanol; 0.328, 0.657, or 0.985 g when *n*-butanol; 0.385, 0.769, or 1.154 g when *n*-octanol; and 0.413, 0.825, or 1.238 when *n*-decanol) and 25 mL of solvent (cyclohexane, hexane, or isooctane) were added to a 125 mL dry round-bottom flask before stirring and heating to 30, 40, 50, or 60 °C. After 48 h of reaction, the lipase was recovered by filtration on a Whatman filter paper number 41 and rinsed with 50 mL hexanes. The filtrate solvent was evaporated under reduced pressure to recover the unreacted carboxylic acids and the (*S*)-ketoprofen ester oily mixture to be then analyzed. To the oily mixture was added to 50 mL of a 10% sodium bicarbonate solution and stirred at 25 °C for 30 min. The reaction mixture (aqueous solution) was extracted with hexane (3 × 30 mL), recovering the non-polar (*S*)-alkyl ketoprofen ester [2]. During all the reactions, samples were removed from the suspensions and monitored at different time intervals by chiral HPLC.

### 4.5. (R)-Ketoprofen Racemization

Several attempts were made in order to ensure a complete racemization of the enriched (*R*)-ketoprofen acid to the 50-50 mixture of (*R,S*)-ketoprofen acid. The first attempt involved enolization under an acidic medium. Then, 10 mmol of enriched (*R*)-ketoprofen 87% ee were added to a 50 mL of 0.1 M H_2_SO_4_ solution and different amounts of DMSO as co-solvents (0%, 1%, 2%, 4%, 8%, 16%) were used [38]. All racemization reactions were carried out in a reflux system for 8 h. Samples were taken by taking an aliquot at different time intervals and extracting the carboxylic acids (ketoprofen) in hexanes to be then analyzed by chiral HPLC by measuring the enantiomeric excess (ee) of the residual carboxylic acids.

The second attempt was performed under alkaline medium. In total, 10 mmol of enriched (*R*)-ketoprofen 87% ee were added to a 50 mL of 0.1 M (NaOH or KOH) solutions, and different amounts of DMSO as co-solvents (0%, 1%, 2%, 4%, 8%, 16%) were used [38]. All racemization reactions were carried out in a reflux system for 12 h. Samples were taken by removing an aliquot and acidifying this to a pH 5.5 (below ketoprofen pKa 5.9) to be then extracted with hexanes and analyzed by chiral HPLC by measuring the enantiomeric excess of the residual carboxylic acids.

The third attempt was carried out using a microwave (CEM Discover Microwave Synthesize) under the following conditions. In total, 2 mmol of enriched (*R*)-ketoprofen 87% ee were added to 20 mL of 0.1 M (H_2_SO_4_, NaOH, or KOH) solutions, and different amounts of DMSO as co-solvents (0%, 1%, 2%, 4%, 8%, 16%) were used. A power of 250 W, medium stirring, and 100 °C were selected as running conditions. The reactions were carried out at different times (15 s, 30 s, 1 min, 3 min, 6 min, and 12 min) which were subsequently acidified in the necessary cases, extracted with hexanes, and analyzed by HPLC to measure enantiomeric excess.

### 4.6. Enzymatic Hydrolysis of Ketoprofen Ester

A typical example is described as follows: 500 mg of (*S*)-decyl ketoprofen ester was added to 20 mL of 50 mM sodium phosphate buffer pH 6.8 containing 100 mg of *Candida rugosa* lipase. Tween 80 surfactant was added to the system with different concentrations (5%, 10%, 15%, and 20%). The mixture was kept at 45 °C in a rotary shaker at 160 rpm [5]. Then, 1 mL samples 1 mL were periodically withdrawn and extracted with 5 mL of hexanes to be analyzed by chiral HPLC.

## 5. Conclusions

Despite significant progress in the field of anti-inflammatory drugs, there is a constant demand for better treatment options with less side effects. In addition to synthesizing new drugs, there is the possibility of eliminating undesired adducts from racemic mixtures; specifically, adducts from commercial, ubiquitous medicines, such as over-the-counter ketoprofen. This manuscript summarized the efforts aimed to develop an environmentally friendly protocol to resolve (*S*)-ketoprofen from the commercial racemic mixture, while eliminating the undesired (*R*)-ketoprofen by converting it to the racemic mixture so it can be recycled. Overall, the presented work presents a proof-of-concept for the kinetic resolution of racemic ketoprofen using *Candida rugosa* lipase as the biocatalyst. To our delight, the conversion and enantioselectivity were excellent for the synthesis of (*S*)-ketoprofen (from 0% to 99% ee) and the racemization of (*R*)-ketoprofen (from 87% to 1.6% ee). Finally, our protocol’s scale was 1.271 g-scale and there is no doubt that it can be further scaled-up to an industrial scale. It is expected that a broad adoption of this methodology should have positive benefits to the consumers as well as the pharmaceutical companies. The authors are currently working on a one-pot dynamic kinetic resolution of ketoprofen, and the outcome should be reported in due time.

## Figures and Tables

**Table 1:** Lipase-catalyzed enantioselective esterification of racemic ketoprofen.

Enz. %	Temp. °C	*Candida rugosa* L.			*Mucor javanicus* L.			*Porcine pancreatic* L.		
		***c%***	**ee**	***E***	***c%***	**ee**	***E***	***c%***	**ee**	***E***
20	30	10.6	52.3	24	11.8	50.9	19	9.9	59.4	22
20	40	22.4	63.5	30	19.4	64.8	40	14.2	66.7	53
20	50	24.7	76.3	28	24.1	70.4	88	17.7	56.2	72
20	60	25.3	42.5	17	21.9	43.0	35	12.8	42.3	31
40	30	17.9	77.9	26	12.4	54.4	19	12.8	62.2	25
40	40	34.8	89.2	54	22.9	72.9	97	18.8	72.1	60
40	50	37.8	92.4	46	34.6	74.9	29	19.5	66.9	66
40	60	36.9	36.4	24	25.2	48.1	27	14.2	41.9	37
60	30	39.7	40.8	83	15.0	50.7	56	13.8	55.4	54
60	40	**43.8**	**87.2**	**165**	25.8	72.9	87	23.9	58.6	62
60	50	42.0	74.8	114	34.9	71.4	101	22.2	45.8	60
60	60	41.4	32.4	23	24.5	68.2	87	12.0	41.6	43

Experimental conditions: 40 °C, stirring at 250 rpm, 48 h reaction time, 5 mmol of racemic ketoprofen, 5 mmol *n*-decanol, and 25 mL of cyclohexane as solvent. Conversion of the esterification reaction (*c*), enantiomeric excess (ee), and enantioselectivity (*E*).

**Table 2:** Alcohol and solvent experimental determination for the resolution of (*S*)-ketoprofen.

Alcohol/Solvent		Hexane	Cyclohexane	Isooctane
***n*-decanol**	*c %*	27.07	43.8	**46.65**
	ee	23.29	87.20	**99**
	*E*	17.64	165	**185**
***n*** **-oc** **tanol**	*c %*	28.22	18.43	26.20
	ee	18.52	16.61	23.92
	*E*	4.76	53.87	26.46
***n*** **-butanol**	*c %*	4.22	3.64	17.48
	ee	0.98	0.78	13.56
	*E*	1.2	1.53	22.67
**methanol**	*c %*	7.19	2.88	8.22
	ee	1.16	0.49	8.02
	*E*	1.35	0.71	3.75

Enantiomeric conversion (*c*), enantiomeric excess (ee) and selectivity (*E*). Reaction conditions: 250 rpm 0.6:1 w:w ratio of enzyme:substrate, 40 °C and 48 h of reaction.

## Data Availability

Data is contained within the article and supplementary files.

## Supplementary Materials

The following are available online at https://www.mdpi.com/article/10.3390/ph14100996/s1, Figure S1: (*R,S*)-ketoprofen FTIR spectrum, Figure S2: (*R,S*)-ketoprofen 1H NMR spectrum, Figure S3: Racemic ketoprofen melting point experiment, Figure S4: HPLC chromatogram of microwave induced racemization, Figure S5: (*S*)-decyl ester ketoprofen FTIR spectrum, Figure S6: (*S*)-decyl ester ketoprofen 1H NMR spectrum, Figure S7: (*S*)-ketoprofen FTIR spectrum, Figure S8: (*S*)-ketoprofen 1H NMR spectrum.

## References

[1] Chavez-Flores D., Salvador J.M. (2012). Facile conversion of racemic ibuprofen to (S)-ibuprofen. Tetrahedron Asymmetry.

[2] Chávez-Flores D., Salvador J.M. (2009). Commercially viable resolution of ibuprofen. Biotechnol. J..

[3] Sardella R., Ianni F., Di Michele A., Di Capua A., Carotti A., Anzini M., Natalini B. (2017). Enantioresolution and stereochemical characterization of two chiral sulfoxides endowed with COX-2 inhibitory activity. Chirality.

[4] Anthonsen T., Hoff B.H. (1998). Resolution of derivatives of 1,2-propanediol with lipase B from Candida antarctica: Effect of substrate structure, medium, water activity and acyl donor on enantiomeric ratio. Chem. Phys. Lipids.

[5] Liu Y.-Y., Xu J.-H., Hu Y. (2000). Enhancing effect of Tween-80 on lipase performance in enantioselective hydrolysis of ketoprofen ester. J. Mol. Catal. B Enzym..

[6] Fernandes C., Palmeira A., Ramos I.I., Carneiro C., Afonso C., Tiritan M.E., Cidade H., Pinto P., Saraiva M.L.M., Reis S. (2017). Chiral Derivatives of Xanthones: Investigation of the Effect of Enantioselectivity on Inhibition of Cyclooxygenases (COX-1 and COX-2) and Binding Interaction with Human Serum Albumin. Pharmaceuticals.

[7] Ríos-Quintana R., Estrada-Hernández L.O. (2018). Descripción y Cuantificación de Riesgos Atribuidos a Analgésicos Antiinflamatorios No Esteroides No Selectivos Consumidos Por La Población Mexicana. Med. Interna México.

[8] Nugrahani I., Parwati R. (2021). Challenges and Progress in Nonsteroidal Anti-Inflammatory Drugs Co-Crystal Development. Molecules.

[9] De Loera-González M.A., Sánchez–Rodríguez S.H., Castro-Pastrana L.I., Flores-de la Torre J.A., López-Luna A. (2016). Ecofarmacovigilancia. Rev. CENIC Cienc. Biológicas.

[10] Matysiak S., Kula J., Błaszczyk A. (2019). Chiral amide derivatives of ricinoleic acid and 3-hydroxynonanoic acid synthesis and cytotoxic activity. Med. Chem. Res..

[11] Huras B., Zakrzewski J., Krawczyk M., Bombińska D., Cieniecka-Rosłonkiewicz A., Michalczyk A. (2017). Synthesis and fungicidal activity of 2-methylalkyl isonicotinates and nicotinates. Med. Chem. Res..

[12] Bui C., Rosenau T., Hettegger H. (2021). Polysaccharide- and β-Cyclodextrin-Based Chiral Selectors for Enantiomer Resolution: Recent Developments and Applications. Molecules.

[13] Ong A., Kamaruddin A., Bhatia S., Long W., Lim S., Kumari R. (2006). Performance of free Candida antarctica lipase B in the enantioselective esterification of (R)-ketoprofen. Enzym. Microb. Technol..

[14] Mwamwitwa K.W., Kaibere R.M., Fimbo A.M., Sabitii W., Ntinginya N.E., Mmbaga B.T., Shewiyo D.H., Shearer M.C., Smith A.D., Kaale E.A. (2020). A retrospective cross-sectional study to determine chirality status of registered medicines in Tanzania. Sci. Rep..

[15] Yuan X., Wang L., Liu G., Dai G., Tang K. (2019). Resolution of (R, S)-ibuprofen catalyzed by immobilized Novozym40086 in organic phase. Chirality.

[16] Huang K., Jiao F., Liu S., Li X., Huang D. (2006). Enantioselective Extraction of Ketoprofen Enantiomers Using Ester Alcohol (R, R)-Di-Tartarates or (S, S)-Di-Tartarates as Chiral Selector. Lat. Am. Appl. Res.

[17] Mokhtari Z., Hekmatdoost A., Nourian M. (2017). Antioxidant efficacy of vitamin D. J. Parathyroid. Diesase.

[18] Foster R., Jamali F., Russell A., Alballa S. (1988). Pharmacokinetics of ketoprofen enantiomers in healthy subjects following single and multiple doses. J. Pharm. Sci..

[19] Calcaterra A., D’Acquarica I. (2018). The market of chiral drugs: Chiral switches versus de novo enantiomerically pure compounds. J. Pharm. Biomed. Anal..

[20] Alkatheeri N.A., Wasfi I.A., Lambert M. (1999). Pharmacokinetics and metabolism of ketoprofen after intravenous and intramuscular administration in camels. J. Veter-Pharmacol. Ther..

[21] Kantor T.G. (1986). Ketoprofen: A Review of Its Pharmacologic and Clinical Properties. Pharmacother. J. Hum. Pharmacol. Drug Ther..

[22] Siodmiak T., Rumiński J.K., Marszall M.P. (2012). Application of Lipases from Candida rugosa in the Enantioselective Esterification of (R,S)-Ibuprofen. Curr. Org. Chem..

[23] Nguyen H.C., Huong D.T.M., Juan H.-Y., Su C.-H., Chien C.-C. (2018). Liquid Lipase-Catalyzed Esterification of Oleic Acid with Methanol for Biodiesel Production in the Presence of Superabsorbent Polymer: Optimization by Using Response Surface Methodology. Energies.

[24] Akanbi T.O., Barrow C.J. (2018). Lipase-Produced Hydroxytyrosyl Eicosapentaenoate is an Excellent Antioxidant for the Stabilization of Omega-3 Bulk Oils, Emulsions and Microcapsules. Molecules.

[25] Sun M., Nie K., Wang F., Deng L. (2020). Optimization of the Lipase-Catalyzed Selective Amidation of Phenylglycinol. Front. Bioeng. Biotechnol..

[26] Dovale-Rosabal G., Rodríguez A., Espinosa A., Barriga A., Aubourg S. (2021). Synthesis of EPA- and DHA-Enriched Structured Acylglycerols at the *sn*-2 Position Starting from Commercial Salmon Oil by Enzymatic Lipase Catalysis under Supercritical Conditions. Molecules.

[27] Wu W.-X., Liu Z. (2020). Novozym 435-Catalyzed Synthesis of Well-Defined Hyperbranched Aliphatic Poly(β-thioether ester). Molecules.

[28] Ramnath L., Sithole B., Govinden R. (2017). Identification of lipolytic enzymes isolated from bacteria indigenous to Eucalyptus wood species for application in the pulping industry. Biotechnol. Rep..

[29] Henke E., Schuster S., Yang H., Bornscheuer U.T. (2000). Lipase-Catalyzed Resolution of Ibuprofen. Biocatalysis.

[30] Liu J., Zhang Y., Qiu L.H., Yang F., Ye L., Xia Y. (2004). Kinetic resolution of ketoprofen ester catalyzed by lipase from a mutant of CBS 5791. J. Ind. Microbiol. Biotechnol..

[31] Cernia E., Delfini M., Di Cocco E., Palocci C., Soro S. (2002). Investigation of lipase-catalysed hydrolysis of naproxen methyl ester: Use of NMR spectroscopy methods to study substrate-enzyme interaction. Bioorganic Chem..

[32] Lin C.-N., Tsai S.-W. (2000). Dynamic kinetic resolution of suprofen thioester via coupled trioctylamine and lipase catalysis. Biotechnol. Bioeng..

[33] Antona N.D., Lombardi P., Nicolosi G., Salvo G. (2002). Large scale preparation of enantiopure S-ketoprofen by biocatalysed kinetic resolution. Process. Biochem..

[34] Allen A.E., MacMillan D.W.C. (2011). Asymmetric Synthesis of (S)-Ketoprofen. Synfacts.

[35] Yao Y., Zou X., Wang Y., Yang H., Ren Z., Guan Z. (2021). Palladium-Catalyzed Asymmetric Markovnikov Hydroxycarbonylation and Hydroalkoxycarbonylation of Vinyl Arenes: Synthesis of 2-Arylpropanoic Acids. Angew. Chem. Int. Ed..

[36] Calmes M., Daunis J., Jacquier R., Natt F. (1994). Asymmetric synthesis of ketoprofen: A surprising base catalyst effect during asymmetric addition of pantolactone to methyl (3-benzoylphenyl) ketene. Tetrahedron.

[37] Ramminger C., Zim D., Lando V.R., Fassina V., Monteiro A.L. (2000). Transition-metal catalyzed synthesis of Ketoprofen. J. Braz. Chem. Soc..

[38] Yuchun X., Huizhou L., Jiayong C. (2000). Kinetics of base catalyzed racemization of ibuprofen enantiomers. Int. J. Pharm..

