# Supplementary material for: Lipase Assisted (S)-Ketoprofen Resolution from Commercially Available Racemic Mixture
Source: Pharmaceuticals (Basel). 2021 Sep 29;14(10):996. doi: 10.3390/ph14100996 (PMC8541352; doi:10.3390/ph14100996)
Supplement: Supplementary file 1 [file pharmaceuticals-14-00996-s001.zip › pharmaceuticals-1392548-supplementary.pdf]

pharmaceuticals

MDPI

Article

Lipase Assisted (*S*)-Ketoprofen Resolution from Commercially Available Racemic Mixture

Daniela Estrada-Valenzuela [1], Víctor H. Ramos-Sánchez [1], Gerardo Zaragoza-Galán [1], Jose C. Espinoza-Hicks [1], Alejandro Bugarin [2,*] and David Chávez-Flores [1,*]

[1] Facultad de Ciencias Químicas, Universidad Autónoma de Chihuahua, Circuito Universitario, Chihuahua. 31125, Mexico; p291826@uach.mx (D.E.-V.), vramos@uach.mx (V.H.R.-S.), gzaragoza@uach.mx (G.Z.-G.), jhicks@uach.mx (J.C.E.-H.)

[2] Department of Chemistry & Physics, Florida Gulf Coast University, Fort Myers, FL, Florida, 33965, USA

* Correspondence: abugarin@fgcu.edu (A.B.) ; dchavezf@uach.mx (D.C.-F.)

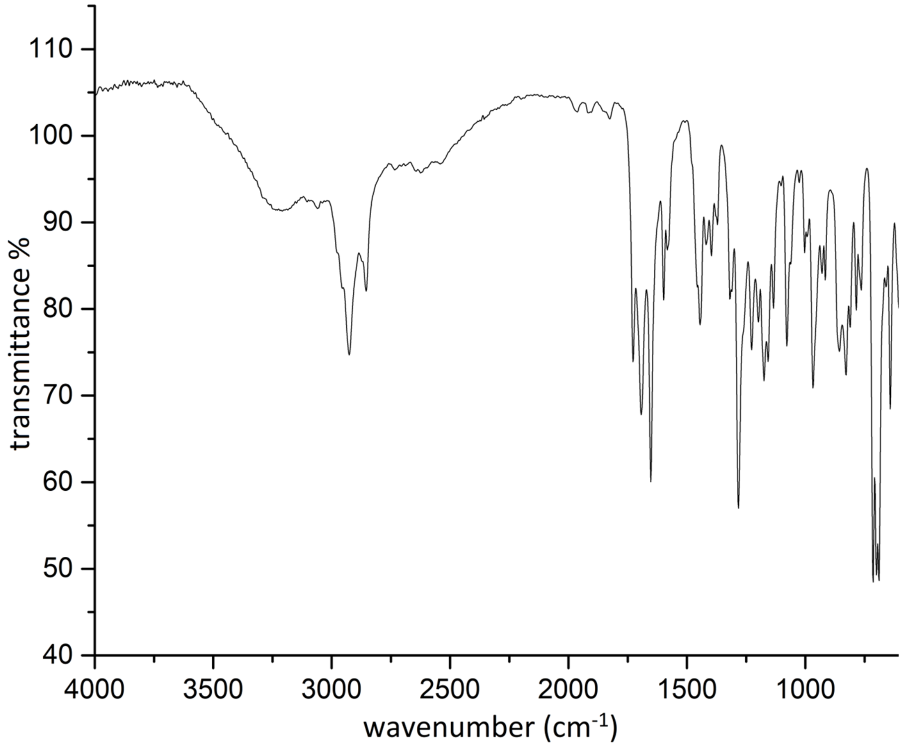

Figure S1. (*R,S*)-Ketoprofen FTIR spectrum.

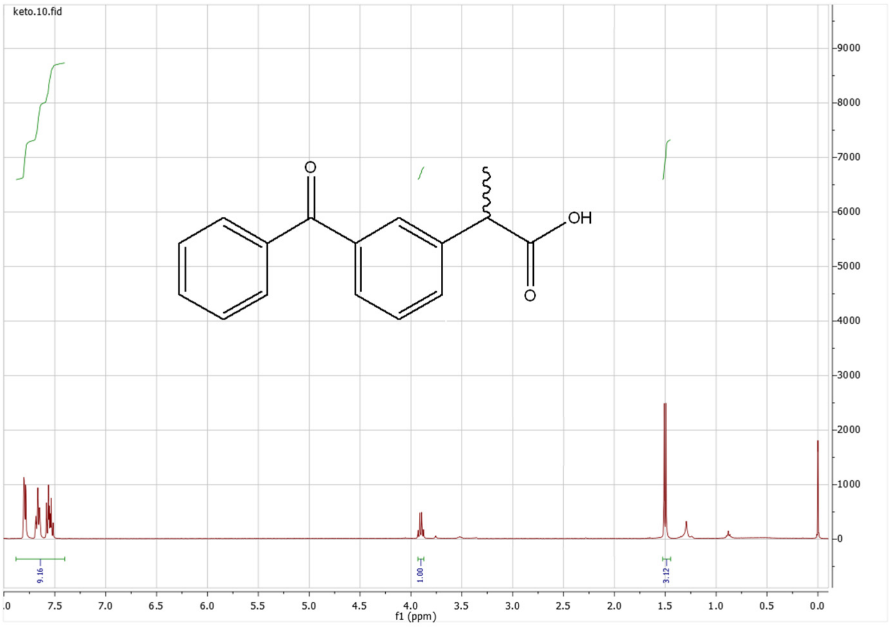

Figure S2. (*R*,*S*)-Ketoprofen ^{1}H NMR spectrum.

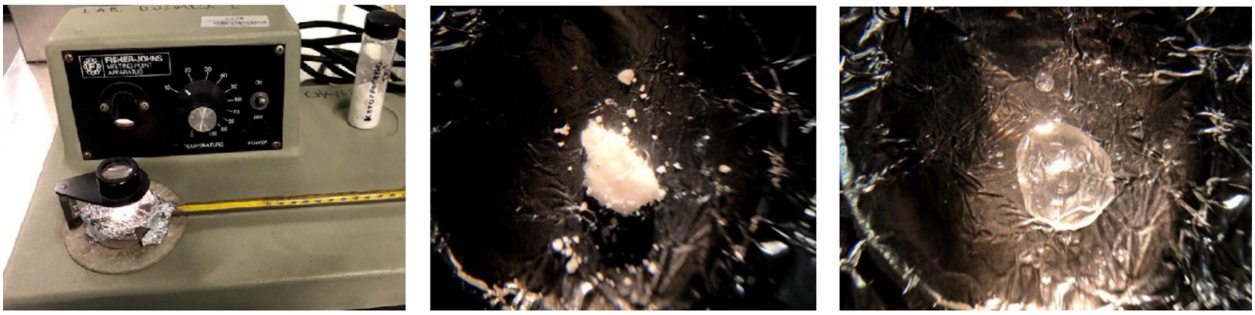

Figure S3. Racemic ketoprofen melting point experiment. (m.p. = 93-94 °C).

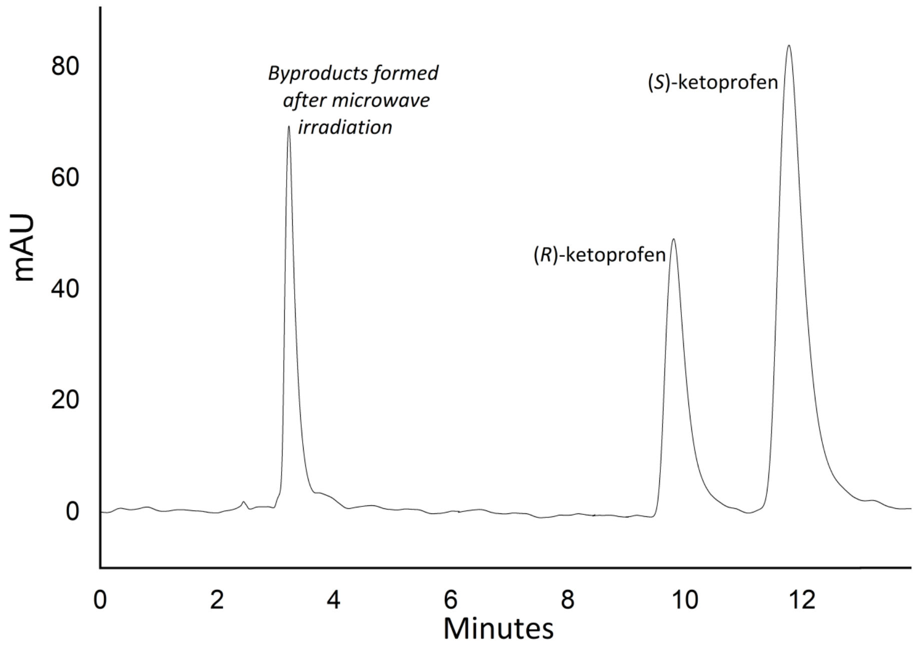

Figure S4. HPLC chromatogram with the evidence of byproduct formation during the microwave racemization attempt. For this experiment, pure (S)-ketoprofen was used as pure enantiomer to study its racemization using microwave as energy source.

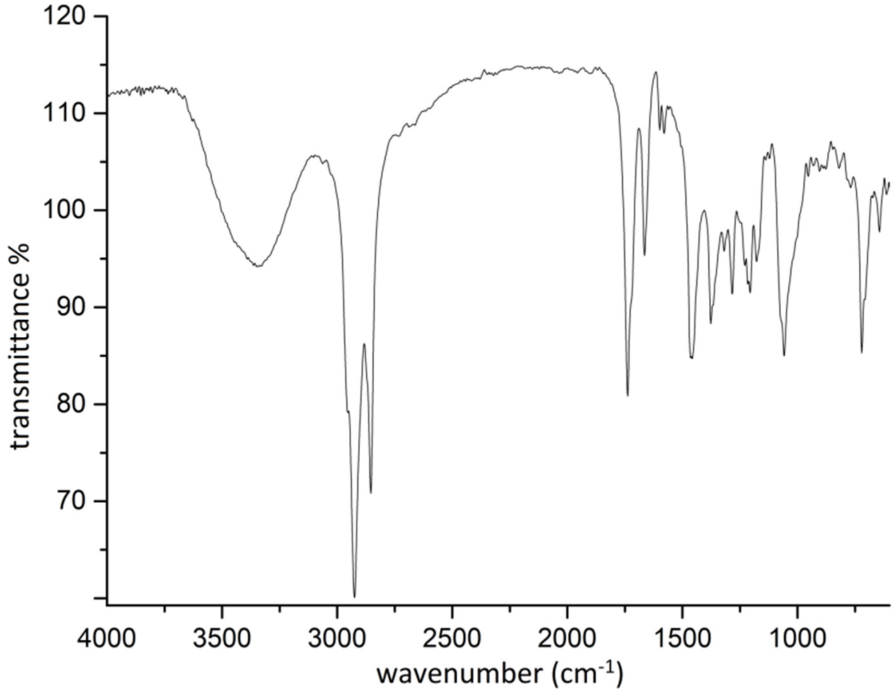

Figure S5. (*S*)-decyl ketoprofen ester FTIR spectrum (dissolved in methanol).

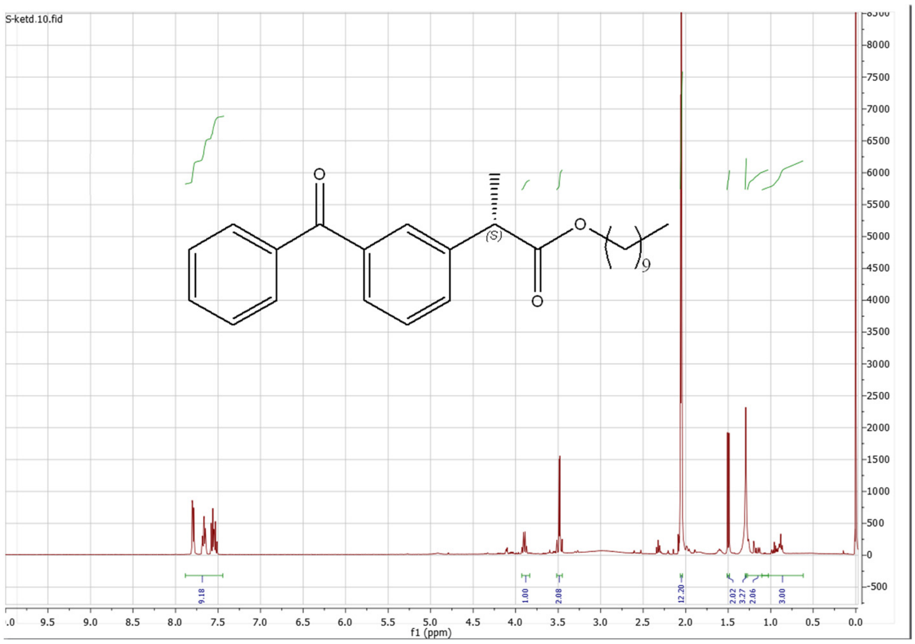

Figure S6. (*S*)-decyl ketoprofen ester ^{1}H NMR spectrum.

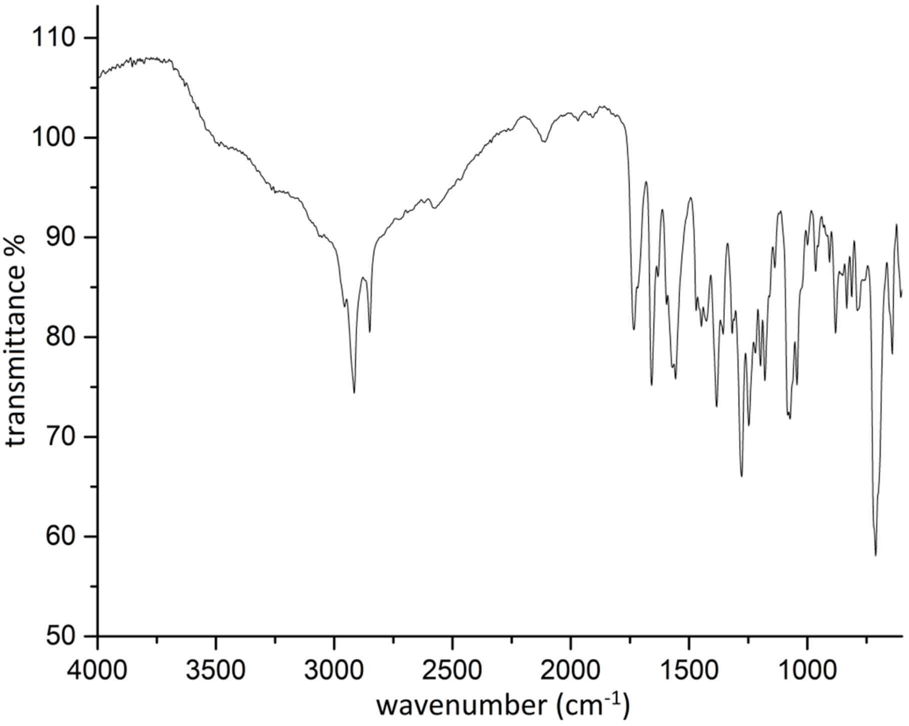

Figure S7. (*S*)-ketoprofen FTIR spectrum.

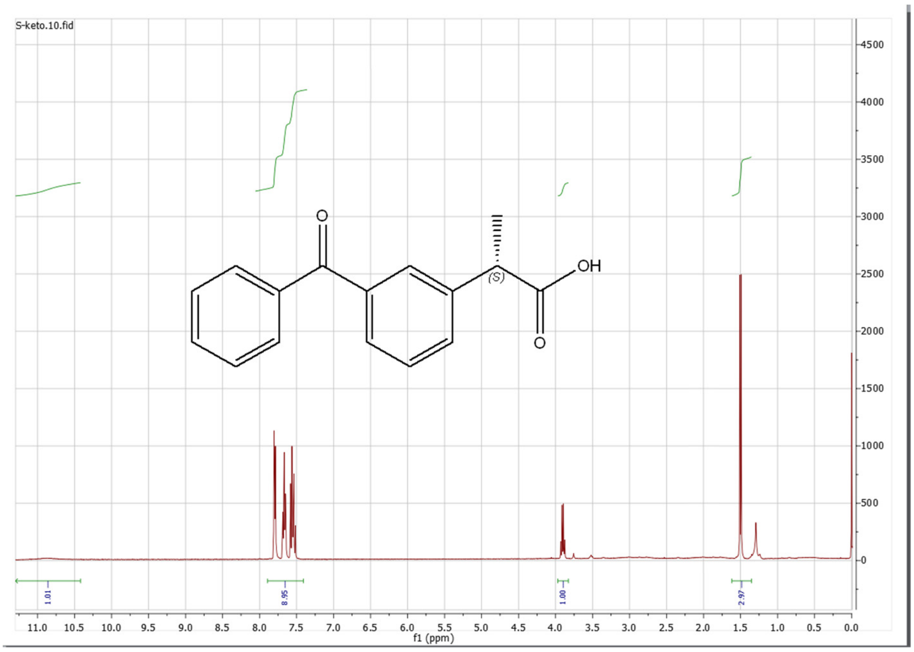

Figure S8. (*S*)-ketoprofen ^{1}H NMR spectrum.
